# Supplementary material for: Uncertainty in the association between socio-demographic characteristics and mental health
Source: PLoS One. 2023 Nov 22;18(11):e0294664. doi: 10.1371/journal.pone.0294664 (PMC10664908; doi:10.1371/journal.pone.0294664)
Supplement: Attachment — Submitted filename: UncertaintySDAndMH-Resub-ResponseToReviewers.docx [file pone.0294664.s001.docx]

**Editor's comments**

|  | **Referee Comment** | **Author's Response** |
| --- | --- | --- |
| 1 | Thank you for submitting your manuscript to PLOS ONE. After careful consideration, we feel that it has merit but does not fully meet PLOS ONE’s publication criteria as it currently stands. Therefore, we invite you to submit a revised version of the manuscript that addresses the points raised during the review process. | We thank the editor for the invitation, giving us the opportunity to submit a revised version of our paper. |
| 2 | I encourage the authors to carefully address the comments from the reviewers and in particular, those made by reviewer 2. I agree that the methodology used should be discussed more in depth and especially the info gap model and its probabilistic assumptions. | Thanks for the explanation and the provided clarifications. |
| 6 | Please submit your revised manuscript by Sep 03 2023 11:59PM | We are submitting our revised manuscript before the deadline. |

**Referee #1 comments**

|  | **Referee Comment** | **Author's Response** | **Revised Text** |
| --- | --- | --- | --- |
| 1 | 1. Is the manuscript technically sound, and do the data support the conclusions?  The manuscript must describe a technically sound piece of scientific research with data that supports the conclusions. Experiments must have been conducted rigorously, with appropriate controls, replication, and sample sizes. The conclusions must be drawn appropriately based on the data presented.  **Reviewer #1: Yes** | Thanks for the positive evaluation. |  |
|  | 2. Has the statistical analysis been performed appropriately and rigorously?  **Reviewer #1: Yes** | Thanks for the positive response. |  |
|  | 3. Have the authors made all data underlying the findings in their manuscript fully available?  The PLOS Data policy requires authors to make all data underlying the findings described in their manuscript fully available without restriction, with rare exception (please refer to the Data Availability Statement in the manuscript PDF file). The data should be provided as part of the manuscript or its supporting information, or deposited to a public repository. For example, in addition to summary statistics, the data points behind means, medians and variance measures should be available. If there are restrictions on publicly sharing data—e.g. participant privacy or use of data from a third party—those must be specified.  **Reviewer #1: No** | We addressed the data issues in the resubmission, according to the PLOS Data policy: The data can made available upon request. |  |
|  | 4. Is the manuscript presented in an intelligible fashion and written in standard English?  PLOS ONE does not copyedit accepted manuscripts, so the language in submitted articles must be clear, correct, and unambiguous. Any typographical or grammatical errors should be corrected at revision, so please note any specific errors here.  **Reviewer #1: No** | We reviewed the manuscript thoroughly. |  |
|  | This is a very interesting piece of work that highlights the importance of a "robustness to deep uncertainty axis" as a third dimension in the consideration of psychometric information where, currently validity in terms of individual and generalizability robustness are generally established. Thus, it is a third dimension to take into account. The authors use a French epidemiological survey about mental health impacts of the Covid pandemic to explore their ideas. They develop a model of info-gap robustness to mental health assessment, showing how the robustness to deep uncertainty axis interacts with the other two axes. They highlight the contributions and the limitations of this approach. | Thanks for the summary and the positive assessment. |  |
|  | I have two suggestions to what seems a valid piece of work. First, to expand the description of "Knightian" uncertainty in the introduction. This concept is crucian to the argument. I had to explore extensively beyond the manuscript to begin to understand this and, had I not been reviewing the paper, might have given up. | We added a detailed and explicit definition of "Knightian" uncertainty in the introduction. | Section 1 (Introduction), immediately before Figure 1. |
|  | Similarly, the authors might consider including the crux of their findings from Tempo in the abstract. This would help the reader to be carried through what is quite complex (for a non-psychometrician) writing. At the moment, the point of the paper is demonstrated only quite late in the manuscript. | We added a short sentence about the research significance and contribution in the abstract. | Abstract |
|  | Readers are unable to replicate the findings. Can the authors say more about data availability and make their code available? | We addressed the data issues in the resubmission, according to the PLOS Data policy: The data can made available upon request. |  |

**Referee #2 comments**

|  | **Referee Comment** | **Author's Response** | **Revised Text** |
| --- | --- | --- | --- |
| 1 | 1. Is the manuscript technically sound, and do the data support the conclusions?  The manuscript must describe a technically sound piece of scientific research with data that supports the conclusions. Experiments must have been conducted rigorously, with appropriate controls, replication, and sample sizes. The conclusions must be drawn appropriately based on the data presented.  **Reviewer #2: Partly** | The comments were addressed shortly in the limitations, according to the editor’s suggestion. | The revised test is located in the “Methodology”, “Results”, and “Conclusions” sections |
|  | 2. Has the statistical analysis been performed appropriately and rigorously?  **Reviewer #2: No** | Thanks for the evaluation. We addressed the concerns raised by the reviewer in our revised version, and hope that this will change the response in the next evaluation. |  |
|  | 3. Have the authors made all data underlying the findings in their manuscript fully available?  The PLOS Data policy requires authors to make all data underlying the findings described in their manuscript fully available without restriction, with rare exception (please refer to the Data Availability Statement in the manuscript PDF file). The data should be provided as part of the manuscript or its supporting information, or deposited to a public repository. For example, in addition to summary statistics, the data points behind means, medians and variance measures should be available. If there are restrictions on publicly sharing data—e.g. participant privacy or use of data from a third party—those must be specified.  **Reviewer #2: No** | We addressed the data issues in the resubmission, according to the PLOS Data policy: The data can made available upon request. |  |
|  | 4. Is the manuscript presented in an intelligible fashion and written in standard English?  PLOS ONE does not copyedit accepted manuscripts, so the language in submitted articles must be clear, correct, and unambiguous. Any typographical or grammatical errors should be corrected at revision, so please note any specific errors here.  **Reviewer #2: No** | We reviewed the manuscript thoroughly. |  |
|  | The paper deals with the development of a new approach to measure robustness in order to deepen uncertainty in the test of association between socio-demographic factors and Mental Health. The analysis of deep uncertainty is carried out using the concept of robustness in info-gap decision theory. Data collected in surveys carried out before and during the COVID-19 pandemic are analysed.  The paper is interesting and cover newsworthy topics from both, the methodological (robustness in info-gap decision theory) and the practical (association between socio-demographic characteristics) points of view. However it cannot be published in the current version. If the following changes are made, it may be suitable for publication. Please, in the revised version, highlight the changes with respect to the original submission. | Thanks for the summary and the overall positive assessment, and the suggestions to improve the manuscript. We did our best to answer the raised concerns, as detailed below. |  |
|  | GENERAL COMMENT  In general, some descriptions of the adopted methods are not clear and sufficient. Furthermore, several choices about methodological aspects are not well justified and some of them are quite questionable. Furthermore, there are errors and inaccuracies in the notations used in the formulas. | We added a detailed and explicit definition of "Knightian" uncertainty in the introduction. For notations, we reviewed thoroughly the manuscript following the reviewer’s recommendations. |  |
|  | SPECIFIC COMMENTS  The literature review about contributions on mental health issues in COVID-19 pandemic is not exhaustive and must be extended. Some examples of interesting papers on the topic are the following:  - Moreno, C., Wykes, T., Galderisi, S., Nordentoft, M., Crossley, N., Jones, N., Cannon, M., Correll, C.U., Byrne, L., Carr, S., et al. (2020). How mental health care should change as a consequence of the COVID‐19 pandemic. Lancet Psychiatry, 7, 813–824. https://doi.org/10.1016/S2215‐0366(20)30307‐2.  - Msherghi, A., Alsuyihili, A., Alsoufi, A., Ashini, A., Alkshik, Z., Alshareea, E., Idheiraj, H., Nagib, T., Abusriwel, M., Mustafa, N., et al. (2021) Mental Health Consequences of Lockdown During the COVID‐19 Pandemic: A Cross-Sectional Study. Front. Psychol. 12, 605279. https://doi.org/10.3389/fpsyg.2021.605279.  - Hervalejo, D., Carcedo, R.J., and Fernández-Rouco, N. (2020) Family and mental health during the confinement due to the COVID‐19 pandemic in Spain: The perspective of the counselors participating in psychological helpline services. J. Comp. Fam. Stud. 51, 399–416. https://doi.org/10.3138/jcfs.51.3‐4.014.  - Bonnini S, Borghesi M (2022), Relationship between Mental Health and Socio‐Economic, Demographic and Environmental Factors in the COVID‐19 Lockdown Period‐A Multivariate Regression Analysis, Mathematics, 10(18), 3237. https://doi.org/10.3390/math10183237  Ahmed, M.Z., Ahmed, O., Aibao, Z., Hanbin, S., Siyu, L., Ahmad, A. (2020) Epidemic of COVID‐19 in China and associated psycho‐logical problems. Asian J. Psychiatry, 51, 102092. https://doi.org/10.1016/j.ajp.2020.102092. | Thanks for the recommendations: We added the papers recommended by the reviewer.  We are aware of the large literature corpus about mental health issues during the COVID-19 pandemic, and our intention was only to show a representative sample. We did not intend to show an extensive sample, nor an exhaustive literature review. In our opinion, the actual sample (enlarged by the papers suggested by the reviewer) is enough for our purposes. | Section 3 (Substantive mental health questions)  Table 2  Section 5 (Discussion and Conclusions) |
|  | page 4, section 2, 5 lines after the title: "We should note that we intentionally chose a simplified statistical technique in order to focus primarily on the robustness analysis...". I understand that the robustness analysis is the main contribution of the study but, in general, you should choose a suitable statistical technique according to the design of the study, the goals, the type of variables, the assumptions and the reliability, and not according to simplicity. Either the statistical technique is suitable or it is not. Please comment on this point and explain better. | Thanks for the comment, indeed, the sentence was not most appropriate, and we corrected it. | Section 2 (Methodology: Individual, collective, and robustness components) |
|  | page 5, 4th line from the top: you used a very unusual notation for p_ij, because usually, in matrix notation, the first subscript i represents the row and the second j the column... | We corrected the matrix notation | Table 1 |
|  | page 5, from 4th line to formula (1): the explanation of the test and of the hypotheses is confused, just to say that you compare the marginal probability distributions of the two populations. Anyway, such definition of the problem is misleading because the test on the association between two categorical variables is conceptually different from the test on the comparison between the probability distributions of two populations. You should change and simplify the problem definition. | The discussion has been clarified, emphasizing that the association is tested in terms of the marginal probability distributions. | Section 2.2 (Collective validity) |
|  | formula (1): p_12=p_22 is un-useful | This second relation in hypothesis H0 indeed derives from the first relation in H0.  It is thus redundant. However, it is useful in the sense of making explicit the assertion entailed in H0. That the second relation derives from the first is acknowledged  in the revised explanation of H0, but the second relation is retained for clarity and  explicitness of H0. | Explanation following Equation 1. |
|  | in order to test the effects of socio-demographic factors you should consider a suitable procedure that jointly take into account all the factors simultaneously. If you consider one factor at a time, you don't test the associations between variables net of the others. Comment on this choice of considering separate chi-square tests, one per socio-demographic factor. | We agree, and added an explanatory paragraph following Equation 1 (starting with “The emphasis of this paper…”) | Section 2.2 (Collective validity) |
|  | page 5, formulas (3) and (4) and later in the paper: the same notation is used to represent the observed and the expected number of observations. Please, distinguish the two quantities. | We agree with the reviewer: This notational distinction was introduced later, but it is already required in equations (3) and (4). Therefore, we added an explanatory paragraph. | Paragraph following Equation 2. |
|  | page 5, 5th line from the bottom: replace "Let Qc(p) denote the value of the chi-square statistic at a level of significance p with one degree of freedom" with "Qc(p) represents the quantile of the chi-square distribution corresponding to a cumulative probability of p". | We prefer not to make the suggested change for the following two reasons. First, the  proposed alternative employs a technical statistical term — quantile — which may not be familiar to the reader and is not essential to this current problem. Second, the proposed alternative does not specify the degree of freedom which is a necessary  parameter in specifying a chi-squared distribution. |  |
|  | page 6, 1st row after formula (5): replace "accepted" with "not rejected" | Done | Last paragraph before section 2.3 (Robustness to uncertainty) |
|  | page 6, from 5th line to the 2nd line the bottom, page 7, formula (6): you state that the info-gap uncertainty does not depend on any probabilistic assumptions, but you base the info-gap model on the assumption that the observations are generated by a Poisson random variable. This is an evident contradiction! What happens if the data are not Poisson distributed? Is there a general formulation that doesn't need the assumption of Poisson distribution? | We definitely do not assert that the observations are Poisson distributed. However,  the sentences at the bottom of p.6 need clarification, which we have implemented. | Section 2.3 (Robustness to uncertainty) |
|  | page 7, 1st and 2nd line after formula (6): "The N_ij only take discrete values, but we are treating them as continuous variables for simplicity". Again, simplicity drives your methodological choices. This is questionable because discrete data should imply the use of discrete variables. You should better justify this choice and motivate the fact that it doesn't adversely affect the reliability of results. | Thanks for pinpointing this detail. We added a paragraph following equation 6, justifying this approximation. | Section 2.3 (Robustness to uncertainty), after Equation 6 |
|  | page 8, 3rd line from the top: why do you consider the inverse function? you should justify this choice and motivate the fact that it doesn't affect the reliability of results. | The discussion following Equation 8 was updated to include an explanation answering the reviewer’s concern. | Section 2.3 (Robustness to uncertainty), after Equation 8 |
|  | page 8, 2nd line to the title of section 3: please clearly indicate which tables are you specifically talking about | We amended the text clarifying that we refer to Table 3. | Last paragraph before Section 3 (Substantive mental health questions) |
|  | page 10, 10th line to the bottom: why do you choose the 85th percentile? please justify this choice | We have justified this choice by adding a short explanation earlier in the text, when the 85^th^ percentile is first mentioned. | Section 2.1 (Individual validity), in the paragraph before Table 1 |
