# Supplementary material for: Uncertainty in the association between socio-demographic characteristics and mental health
Source: PLoS One. 2023 Nov 22;18(11):e0294664. doi: 10.1371/journal.pone.0294664 (PMC10664908; doi:10.1371/journal.pone.0294664)
Supplement: Attachment — Submitted filename: UncertaintySDAndMH-Resub2-ResponseToReviewers.docx [file pone.0294664.s002.docx]

**Editor's comments**

|  | **Referee Comment** | **Author's Response** |
| --- | --- | --- |
| 1 | Thank you for submitting your manuscript to PLOS ONE. After careful consideration, we feel that it has merit but does not fully meet PLOS ONE’s publication criteria as it currently stands. Therefore, we invite you to submit a revised version of the manuscript that addresses the points raised during the review process. | We thank the editor for the invitation, giving us the opportunity to submit a revised version of our paper. |
| 2 | Please submit your revised manuscript by Nov 25 2023 11:59PM. | We are submitting our revised manuscript before the deadline. |

**Referee #1 comments**

|  | **Referee Comment** | | **Author's Response** | | | **Revised Text** |
| --- | --- | --- | --- | --- | --- | --- |
| 1 | 1. If the authors have adequately addressed your comments raised in a previous round of review and you feel that this manuscript is now acceptable for publication, you may indicate that here to bypass the “Comments to the Author” section, enter your conflict of interest statement in the “Confidential to Editor” section, and submit your "Accept" recommendation.  **Reviewer #1: All comments have been addressed** | | Thanks for the positive evaluation. | | |  |
|  | 2. Is the manuscript technically sound, and do the data support the conclusions?  The manuscript must describe a technically sound piece of scientific research with data that supports the conclusions. Experiments must have been conducted rigorously, with appropriate controls, replication, and sample sizes. The conclusions must be drawn appropriately based on the data presented.  **Reviewer #1: Yes** | | Thanks for the positive response. | | |  |
| 3 | | 3. Has the statistical analysis been performed appropriately and rigorously?  **Reviewer #1: Yes** | | Thanks for the positive response. |  | |
| 4 | 4. Have the authors made all data underlying the findings in their manuscript fully available?  The PLOS Data policy requires authors to make all data underlying the findings described in their manuscript fully available without restriction, with rare exception (please refer to the Data Availability Statement in the manuscript PDF file). The data should be provided as part of the manuscript or its supporting information, or deposited to a public repository. For example, in addition to summary statistics, the data points behind means, medians and variance measures should be available. If there are restrictions on publicly sharing data—e.g. participant privacy or use of data from a third party—those must be specified.  **Reviewer #1: Yes** | | Thanks for the positive response. | | |  |
| 5 | 5. Is the manuscript presented in an intelligible fashion and written in standard English?  PLOS ONE does not copyedit accepted manuscripts, so the language in submitted articles must be clear, correct, and unambiguous. Any typographical or grammatical errors should be corrected at revision, so please note any specific errors here.  **Reviewer #1: Yes** | | Thanks for the positive response. | | |  |
| 6 | 6. Review Comments to the Author  Please use the space provided to explain your answers to the questions above. You may also include additional comments for the author, including concerns about dual publication, research ethics, or publication ethics. (Please upload your review as an attachment if it exceeds 20,000 characters)  **Reviewer #1: The authors have done a very good job at addressing both sets of reviewer comments. The description of the phenomenon in question is now very useful and the the ms is much more accessible. I like the catastrophe-like figure. I hope the work gets some traction in the field.** | | Thanks for the comments, the summary, and the positive assessment. | | |  |
| 7 | 7. PLOS authors have the option to publish the peer review history of their article (what does this mean?). If published, this will include your full peer review and any attached files.  If you choose “no”, your identity will remain anonymous, but your review may still be made public.  Do you want your identity to be public for this peer review? For information about this choice, including consent withdrawal, please see our Privacy Policy.  **Reviewer #1: Yes: Peter B. Jones** | | Thanks again to Peter B. Jones for the work invested in reviewing the paper, the comments, and the suggestions that helped to improve our original manuscript. | | |  |

**Referee #2 comments**

|  | **Referee Comment** | **Author's Response** | **Revised Text** |
| --- | --- | --- | --- |
| 1 | 1. If the authors have adequately addressed your comments raised in a previous round of review and you feel that this manuscript is now acceptable for publication, you may indicate that here to bypass the “Comments to the Author” section, enter your conflict of interest statement in the “Confidential to Editor” section, and submit your "Accept" recommendation.  **Reviewer #2: (No Response)** | The reviewer’s comments were addressed. A full description is included in point (6) below. |  |
| 2 | 2. Is the manuscript technically sound, and do the data support the conclusions?  The manuscript must describe a technically sound piece of scientific research with data that supports the conclusions. Experiments must have been conducted rigorously, with appropriate controls, replication, and sample sizes. The conclusions must be drawn appropriately based on the data presented.  **Reviewer #2: Yes** | Thanks for the positive response. |  |
| 3 | 3. Has the statistical analysis been performed appropriately and rigorously?  **Reviewer #2: Yes** | Thanks for the positive response. |  |
| 4 | 4. Have the authors made all data underlying the findings in their manuscript fully available?  The PLOS Data policy requires authors to make all data underlying the findings described in their manuscript fully available without restriction, with rare exception (please refer to the Data Availability Statement in the manuscript PDF file). The data should be provided as part of the manuscript or its supporting information, or deposited to a public repository. For example, in addition to summary statistics, the data points behind means, medians and variance measures should be available. If there are restrictions on publicly sharing data—e.g. participant privacy or use of data from a third party—those must be specified.  **Reviewer #2: Yes** | Thanks for the positive response. |  |
| 5 | 5. Is the manuscript presented in an intelligible fashion and written in standard English?  PLOS ONE does not copyedit accepted manuscripts, so the language in submitted articles must be clear, correct, and unambiguous. Any typographical or grammatical errors should be corrected at revision, so please note any specific errors here.  **Reviewer #2: Yes** | Thanks for the positive response. |  |
| 6 | 6. Review Comments to the Author  Please use the space provided to explain your answers to the questions above. You may also include additional comments for the author, including concerns about dual publication, research ethics, or publication ethics. (Please upload your review as an attachment if it exceeds 20,000 characters)  **Reviewer #2: All the suggestions of the previous review have been taken into account except the following:**  **Page 5, 5th line from the bottom: replace "*Let Qc(p) denote the value of the chi-square statistic at a level of significance p with one degree of freedom*" with "*Qc(p) represents the quantile of the chi-square distribution***  ***corresponding to a cumulative probability of p*”.**  **Of course, you are not obliged to use the suggested sentence, but you must change the original one because it is not correct. The value of a statistic is only function of data, neither of the significance level nor of the degrees of freedom. The degrees of freedom and the value of p, concerns a probability distribution. Your sentence makes no sense.**  **In my opinion, if also this point is addressed, the paper is suitable for publication in Plos One.** | We addressed the concerns raised by the reviewer in our revised version, using the suggested sentence, and also adapted the following phrase. | Text between Equations (4) and (5) in section 2.2. (“Collective validity: Chi-Square test for assessing the ‘mental health – socio-demographic characteristics’ associations”) |
| 7 | 7. PLOS authors have the option to publish the peer review history of their article (what does this mean?). If published, this will include your full peer review and any attached files.  If you choose “no”, your identity will remain anonymous, but your review may still be made public.  Do you want your identity to be public for this peer review? For information about this choice, including consent withdrawal, please see our Privacy Policy.  **Reviewer #2: No** | Thanks again to the anonymous reviewer for the work invested in reviewing the paper, the comments, and the suggestions that helped to improve our original manuscript. |  |
