# Supplementary material for: Uncertainty in the association between socio-demographic characteristics and mental health
Source: PLoS One. 2023 Nov 22;18(11):e0294664. doi: 10.1371/journal.pone.0294664 (PMC10664908; doi:10.1371/journal.pone.0294664)
Supplement: Attachment — Submitted filename: UncertaintySDAndMH-Resub3-ResponseToReviewers.docx [file pone.0294664.s003.docx]

**Editor's comments**

|  | **Referee Comment** | **Author's Response** |
| --- | --- | --- |
| 1 | Thank you for submitting your manuscript to PLOS ONE. After careful consideration, we feel that it has merit but does not fully meet PLOS ONE’s publication criteria as it currently stands. Therefore, we invite you to submit a revised version of the manuscript that addresses the points raised during the review process. | We thank the editor for the invitation, giving us the opportunity to submit a revised version of our paper. |
| 2 | The authors are strongly advised to check the formatting of the manuscript in order to allow the reviewers to do their job. In particular, they should double check all the equations and make sure that nothing is lost in the conversion of the manuscript to different file formats. | We addressed the concerns raised by the reviewer in our revised version. We think this is caused by a problem during the conversion from Word to PDF in the Editorial Manager system. We uploaded an additional PDF file, created using our own “Print as PDF file” function in Word, showing how the equations and the text looks. |
| 3 | Please submit your revised manuscript by Dec 08 2023 11:59PM. | We are submitting our revised manuscript before the deadline. |

**Referee #2 comments**

|  | **Referee Comment** | **Author's Response** | **Revised Text** |
| --- | --- | --- | --- |
| 1 | 1. If the authors have adequately addressed your comments raised in a previous round of review and you feel that this manuscript is now acceptable for publication, you may indicate that here to bypass the “Comments to the Author” section, enter your conflict of interest statement in the “Confidential to Editor” section, and submit your "Accept" recommendation.  **Reviewer #2: All comments have been addressed** | Thanks for the positive response. |  |
| 2 | 2. Is the manuscript technically sound, and do the data support the conclusions?  The manuscript must describe a technically sound piece of scientific research with data that supports the conclusions. Experiments must have been conducted rigorously, with appropriate controls, replication, and sample sizes. The conclusions must be drawn appropriately based on the data presented.  **Reviewer #2: Yes** | Thanks for the positive response. |  |
| 3 | 3. Has the statistical analysis been performed appropriately and rigorously?  **Reviewer #2: Yes** | Thanks for the positive response. |  |
| 4 | 4. Have the authors made all data underlying the findings in their manuscript fully available?  The PLOS Data policy requires authors to make all data underlying the findings described in their manuscript fully available without restriction, with rare exception (please refer to the Data Availability Statement in the manuscript PDF file). The data should be provided as part of the manuscript or its supporting information, or deposited to a public repository. For example, in addition to summary statistics, the data points behind means, medians and variance measures should be available. If there are restrictions on publicly sharing data—e.g. participant privacy or use of data from a third party—those must be specified.  **Reviewer #2: Yes** | Thanks for the positive response. |  |
| 5 | 5. Is the manuscript presented in an intelligible fashion and written in standard English?  PLOS ONE does not copyedit accepted manuscripts, so the language in submitted articles must be clear, correct, and unambiguous. Any typographical or grammatical errors should be corrected at revision, so please note any specific errors here.  **Reviewer #2: Yes** | Thanks for the positive response. |  |
| 6 | 6. Review Comments to the Author  Please use the space provided to explain your answers to the questions above. You may also include additional comments for the author, including concerns about dual publication, research ethics, or publication ethics. (Please upload your review as an attachment if it exceeds 20,000 characters)  **Reviewer #2: In my opinion the paper is now almost suitable for publication. To make it suitable, you should solve a formal problem. In equations 7 and 8, and in parts of the text, I see rectangles but probably this is not the right symbol the authors wanted to choose...please check and correct.** | We addressed the concerns raised by the reviewer in our revised version. The equations that caused the edit issues pinpointed by the reviewer were rewritten. | Equations (7) and (8) in section 2.2, and the following paragraph.  In addition, expressions in the fourth paragraph in section 4.2. |
| 7 | 7. PLOS authors have the option to publish the peer review history of their article (what does this mean?). If published, this will include your full peer review and any attached files.  If you choose “no”, your identity will remain anonymous, but your review may still be made public.  Do you want your identity to be public for this peer review? For information about this choice, including consent withdrawal, please see our Privacy Policy.  **Reviewer #2: No** | Thanks again to the anonymous reviewer for the work invested in reviewing the paper, the comments, and the suggestions that helped to improve our original manuscript. |  |
